# Oral Semaglutide in Routine Clinical Practice: Characteristics of People with Type 2 Diabetes Started on the Drug and Changes in Their Clinical Parameters after 24 Weeks of Treatment

**DOI:** 10.3390/jcm13113054

**Published:** 2024-05-23

**Authors:** Maria Pompea Antonia Baldassarre, Giulia Di Dalmazi, Sara Coluzzi, Federica Carrieri, Fabrizio Febo, Giorgia Centorame, Piergiuseppe Cassino, Luigi Piacentino, Marco Giorgio Baroni, Agostino Consoli, Gloria Formoso

**Affiliations:** 1 Department of Medicine and Aging Sciences, “G. d’Annunzio” University of Chieti-Pescara, 66100 Chieti, Italy; maria.baldassarre@unich.it (M.P.A.B.); federica.carrieri@phd.unich.it (F.C.); giorgia.centorame@unich.it (G.C.); piergiuseppe.cassino@gmail.com (P.C.); ludimapi@hotmail.it (L.P.); consoli@unich.it (A.C.); 2 Center for Advanced Studies and Technology (CAST), “G. d’Annunzio” University of Chieti-Pescara, 66100 Chieti, Italy; 3 Endocrinology and Metabolic Disease Clinic of Pescara, 65100 Pescara, Italy; giulia.didalmazi@asl.pe.it (G.D.D.); sara.coluzzi@asl.pe.it (S.C.); fabrizio.febo@asl.pe.it (F.F.); 4 Andrology and Diabetes, Department of Clinical Medicine, Public Health, Life and Environmental Sciences (MeSVA), University of L’Aquila, 67100 L’Aquila, Italy; marcogiorgio.baroni@univaq.it

**Keywords:** type 2 diabetes, GLP1 receptor agonist, oral semaglutide, cardiovascular risk factors, clinical practice, real-world evidence

## Abstract

**Background/Objectives**: Semaglutide is the unique once-daily oral glucagon-like receptor agonist presently available. Aims of this study were to describe clinical characteristics of patients with type 2 diabetes (T2D) initiating oral semaglutide, to assess its effects on glycemic control, body weight (BW) and its tolerability in routine clinical practice. **Methods**: Electronic medical records from two Italian diabetes clinics were evaluated. Mean glycated hemoglobin (HbA1c) and BW were assessed in adults with T2D before and 6 months after oral semaglutide prescription. Treatment discontinuation and safety data were reported. **Results**: A total of 192 patients initiating oral semaglutide (44% female) presented a mean age of 66 years, a diabetes duration of 10 years, HbA1c of 7.9% and a BW of 82.6 kg. Almost 50% of patients were obese. Mean HbA1c and BW changes from baseline to follow up were −0.7% and −2.6 kg, respectively. Greater HbA1c reduction was observed in patients with baseline HbA1c ≥ 8% and with diabetes duration <5 years. The composite endpoint of HbA1c ≤7% and a weight loss ≥5% was achieved in 22.5% of the participants. A total of 40 patients (20.8%) discontinued treatment: 26 because of gastrointestinal adverse events, and 10 due to limited effectiveness in lowering HbA1c and/or BW. **Conclusions:** In a real clinical setting, patients initiating oral semaglutide showed suboptimal metabolic control, short diabetes duration and obesity; a significant improvement in HbA1c and BW was achieved mainly in patients with a more recent diabetes diagnosis, supporting the use of oral semaglutide in the early phase of the disease.

## 1. Introduction

Semaglutide is the first and unique glucagon-like peptide-1 receptor agonist (GLP-1RA) available in oral formulation. The efficacy of oral semaglutide on glycemic control and body weight (BW) reduction as well as drug safety have been consistently demonstrated in the PIONEER trials program which consists of 10 randomized controlled trials conducted on 9543 subjects with type 2 diabetes (T2D) [1,2,3,4,5,6,7,8,9,10]. The PIONEER program was designed to test oral semaglutide in patients at different stages across diabetes natural history (mean diabetes duration, 3.5–15 years) and background treatments (monotherapy, added to 1 or 2 oral glucose-lowering agents, or added to insulin). Based on the PIONEER program studies, oral semaglutide was approved for clinical use by the FDA in 2019 and by the EMA in 2020; the molecule is available in Italy since September 2021 [11].

The American and European guidelines for the management of hyperglycemia in T2D in adults published on 2022 state that a medication with proven cardiovascular benefits should be prescribed to all patients with diabetes at high or very high cardiovascular risk (CVD) regardless of glycated hemoglobin (HbA1c) level [12]. Although the results of the PIONEER 6 study [10] point toward the efficacy of oral semaglutide in reducing CV risk, the evidence from the large, specific cardiovascular outcome trial (CVOT) with this molecule (SOUL study) [13] are not available at present. However, on basis of the available data, it is reasonable to assume that semaglutide treatment is effective in reducing CV risk regardless of the route of administration (oral or subcutaneous) [14]. With oral semaglutide the first GLP-1RA available in oral formulation, physicians’ attitudes toward the positioning of this class of drugs in routine clinical practice needs to be investigated. As a matter of fact, limited data are available about oral semaglutide real-world use.

The aim of this study was therefore to describe the baseline characteristics of patients initiating oral semaglutide in the first year after marketing in two diabetes centers in Italy. The effectiveness and tolerability in a routine clinical setting after 6 months of treatment were also assessed.

## 2. Patients and Methods

This was a retrospective real-world cohort study conducted in two university-based diabetes centers in Italy (Endocrinology and Metabolic Disease Clinic of Pescara and Andrology and Diabetes Unit, S. Salvatore Regional Hospital, L’Aquila, Italy), based on data routinely registered in an electronic chart system software (Metaclinic/METEDA, version 10.14.2.0, San Benedetto del Tronto, AP, Italy) created to assist Italian diabetes outpatient clinics in managing patient data. This study adheres to the tenets of the Declaration of Helsinki and has received approval by the local ethics committee (Internal Review Board, Università degli Studi dell’Aquila protocol number 48,741, on 8 May 2023). Adults with T2D and documented HbA1c and body weight values before oral semaglutide treatment were included. Patients with type 1, secondary, or gestational diabetes or patients missing baseline or follow-up data were excluded. No other exclusion criteria were considered.

The following clinical and laboratory data were collected: age, gender, disease duration, height, body weight (BW), waist circumference (WC), HbA1c level, fasting plasma glucose (FPG), systolic blood pressure (SBP), diastolic blood pressure (DBP), triglycerides, total cholesterol, high-density lipoprotein cholesterol (HDL), low-density lipoprotein cholesterol (LDL), serum creatinine, aspartate aminotransferase (AST), alanine aminotransferase (ALT), gamma-glutamyl transferase (gamma-GT), estimated glomerular filtration rate (eGFR), microalbuminuria and background diabetes therapy. Information on diabetes complications, comorbidities, and concurrent use of concomitant medications were also recorded.

Microangiopathy was defined as the presence of at least one of the following complications: neuropathy, retinopathy, macular oedema, chronic kidney disease, or microalbuminuria. Macroangiopathy was defined as the presence of any of the following: a history of stroke/transient ischemic attack, a history of myocardial infarction, ischemic heart disease, coronary artery disease, coronary revascularization, peripheral arterial disease, and peripheral revascularization.

Oral semaglutide was prescribed during an outpatient clinic visit. It was initiated with a 4-week dose-escalation regimen, starting with the lowest dose of 3 mg, followed by 7 mg; according to the European Summary of Product Characteristics (SPC), the dose escalation to 14 mg was applied in order to achieve further improvement in glycemic control, if tolerated by the patients. Patients were provided with specific advice regarding the appropriate drug management. Instructions emphasized minimal water intake during ingestion, timing the medication half an hour before meals or other drug intake, and ensuring a fasting period of at least 4 h.

Updated values of HbA1c and body weight, adverse events and reasons for the eventual oral semaglutide discontinuation were recorded during follow-up visits after 6 months, according to routine practice of the diabetes centers.

Continuous variables were expressed as the mean and standard deviation (SD), or as the median and interquartile range (IQR) if non-normally distributed, whereas categorical data were presented as the absolute frequency and percentages.

Longitudinal linear mixed models for repeated measures were applied to assess trends over time in continuous endpoints (HbA1c, body wight, WC, SBP, DBP, total cholesterol, HDL cholesterol, triglycerides, LDL cholesterol, AST, ALT, gamma-GT, eGFR, and microalbuminuria). Results were expressed as the estimated mean change from baseline with their 95% confidence intervals (95% C.I.). Statistical analysis was performed on data available at baseline and after 6 months of follow up for each patient.

The mean change in HbA1c and body weight (co-primary outcomes) was assessed on the overall population and stratified by diabetes duration, sex, age, baseline BMI, baseline eGFR, and previous use of dipeptidyl peptidase 4 inhibitors (DPP4i).

Furthermore, the study population was divided into terciles according to baseline HbA1c levels and body weight to investigate their impact on mean change in HbA1c and body weight at follow up, respectively. Comparison between multiple groups (tercile analysis) was performed by the Kruskal–Wallis test.

Statistical significance was defined as *p* < 0.05. Data were analyzed using the statistical package, SAS software (release 9.4—Cary, NC, USA).

## 3. Results

### 3.1. Subjects’ Characteristics

A total of 192 patients with T2D prescribed oral semaglutide between September 2021 and December 2022 were included in this study. Baseline patients’ characteristics are reported in Table 1.

Overall, 100% of patients were Caucasian; 44% of them were women; median (IQR) age and diabetes duration were 67 (15) years and 9 (12) years, respectively. The mean HbA1c was 7.9% (1.2%) (63 ± 13 mmol/mol), whereas the median fasting glucose value was 146 (42.5) mg/dL, 8.1 (2.4) mmol/L.

**Figure 1:**
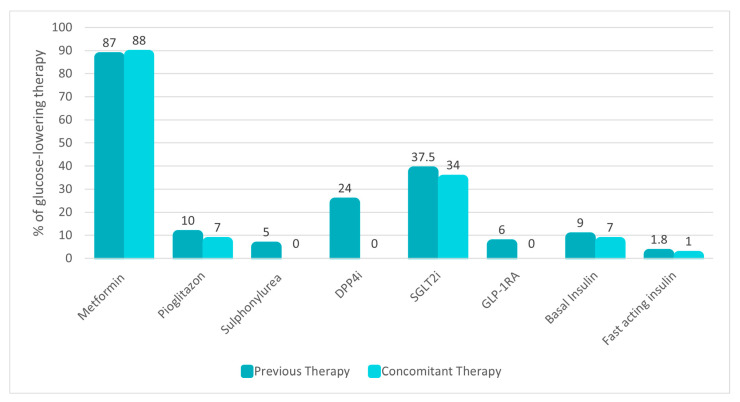
Background glucose-lowering therapy before and after oral semaglutide prescription. Data are presented as the percentage (%) of use in the study population. Abbreviations: DPP4i, dipeptidyl peptidase 4 inhibitors; sodium–glucose cotransporter-2 inhibitors, SGLT2i; GLP-1RA, glucagon-like peptide 1 receptor agonists.

Almost all patients were already treated with a glucose-lowering therapy, specifically metformin (87%), sodium–glucose cotransporter-2 inhibitors (SGLT2i) (37.5%), DPP4i (24%), pioglitazone (10%), basal or fast-acting insulin (9% and 1.8%, respectively), GLP-1RA (6%) and sulfonylureas (5%) (Figure 1).

Patients’ most frequent comorbidities were dyslipidemia (65%), arterial hypertension (63%) and obesity (47%). The main chronic diabetes-related complication were macroangiopathy (31%), and microangiopathy 35% (specifically 25% nephropathy, 16% retinopathy and only 3% neuropathy; Table 1).

Concomitant medication therapy is described in Table 1. The most common therapies were statins (53%) and antihypertensive agents (angiotensin-converting enzyme/angiotensin receptor blockers 48%, beta blockers 35%, calcium blockers 23% and diuretics 23%).

### 3.2. Changes in Metabolic Parameters from Baseline to 6 Months

Changes in metabolic parameters are shown in Table 2. Out of 192 patients, 12 discontinued oral semaglutide before the first follow up because of early interruption (up to 45 days). Effectiveness analysis was conducted on 180 subjects. Overall, after 6 months, mean change in HbA1c was −0.68% (95% C.I. −0.92; −0.43, *p* < 0.0001), and mean change in body weight was −2.63 Kg (95% C.I. −3.37; −1.9, *p* < 0.0001).

**Figure 2:**
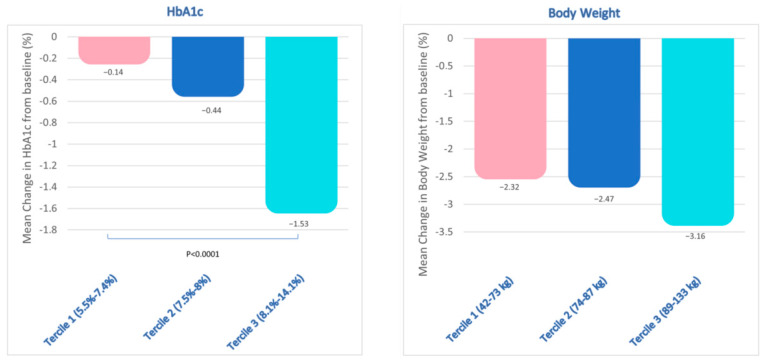
Study population divided in terciles based on the baseline HbA1c (on the **left**) and body weight (on the **right**) levels. Baseline HbA1c terciles were: tercile 1 (5.5–7.4%; *n* = 71); tercile 2 (7.5–8%; *n* = 62); tercile 3 (8.1–14.1%; *n* = 59). *p*= non-significant (n.s.) between HbA1c tercile 1 and tercile 2; *p* < 0.0001 between HbA1c tertile 3 and tertile 1. Baseline body weight terciles were: tercile 1 (42–73 kg; *n* = 54); tercile 2 (74–87 kg; *n* = 53); tercile 3 (89–133 kg; *n* = 51). Among body weight terciles, *p* = n.s.

To assess the impact of baseline HbA1c levels on the mean change in HbA1c at follow up, the study cohort was categorized into three distinct terciles based on baseline HbA1c levels. Tercile 1 (5.5–7.4%) exhibited a mean reduction of −0.14%, tercile 2 (7.5–8%) demonstrated a reduction of −0.44%, while tercile 3 (8.1–14.1%) displayed the most significant reduction of −1.53% (*p* < 0.0001 versus tercile 1) (Figure 2).

Similarly, the study cohort was stratified into three terciles based on the baseline BW (tercile 1: 42–73 kg; tercile 2: 74–87 kg; and tercile 3: 89–133 kg) to assess the impact of baseline BW on the mean reduction in BW at follow up. This analysis performed did not reveal any statistically significant variations in BW reduction across terciles (Figure 2).

**Figure 3:**
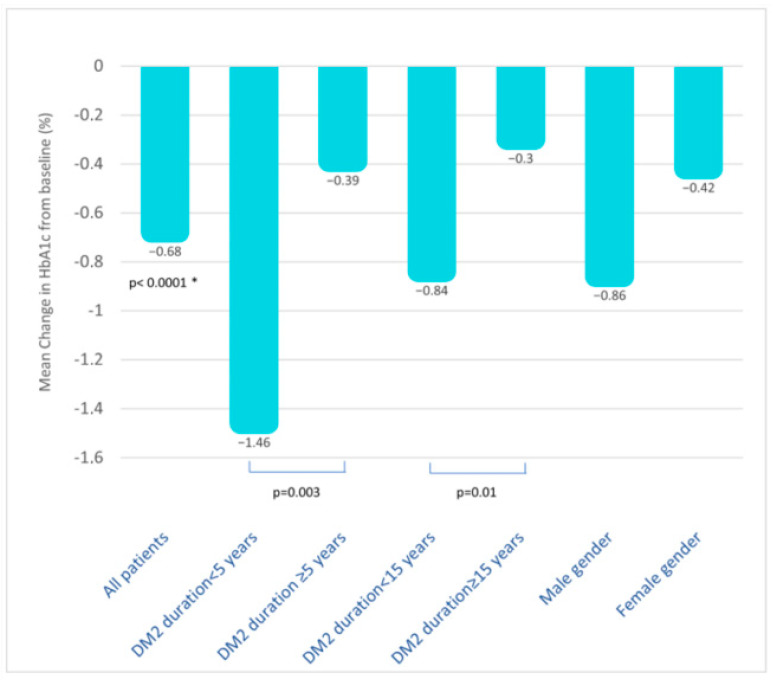
Mean change in HbA1c from baseline (%) in all patients and in subjects stratified by disease duration and gender. Data about stratification by age, BMI categories, eGFR, and previous use of DDP4i are not shown but described in the text. *, *p* value versus baseline. Abbreviations: T2D, type 2 diabetes; BMI, body mass index; eGFR, estimated glomerular filtration rate; DPP4i, dipeptidyl peptidase 4 inhibitors.

Forty-five (45)% of participants successfully achieved an HbA1c target below 7%; concurrently, over the same period of time, 34% of patients obtained body weight loss >5%. Interestingly, 22.5% of the participants achieved the composite outcome of HbA1c levels < 7% and a weight loss > 5%,

Stratification by disease duration revealed that patients with a greater reduction in HbA1c were those with a shorter disease duration (T2D duration < 5 years: −1.46%; 95% C.I. −2.15; −0.77 versus T2D duration ≥ 5 years: −0.39%; 95% C.I. −0.58; −0.19; *p* = 0.003); however, the effectiveness persisted, albeit slightly attenuated, in patients with a disease duration < 15 years (T2D duration < 15 years: −0.84%; 95% C.I. −1.16; −0.51 versus T2D duration ≥ 15 years: −0.30%; 95% C.I. −0.59; −0.20; *p* = 0.01) (Figure 3).

Effective reduction in HbA1c persisted stratifying by age (<65 years: −0.77%, 95% C.I. −1.33; −0.21 and ≥65 years: −0.62%, 95% C.I. −0.86; −0.39), BMI categories (BMI < 25: −0.78%, 95% C.I. −1.64; 0.07; BMI 25–30: −0.75%, 95% C.I. −1.22; −0.27; BMI ≥ 30: −0.74%, 95% C.I. −1.15; −0.34), and eGFR (<60 mL/min/m^2^: −0.72%, 95% C.I. −1.01; −0.41; >60 mL/min/m^2^: −0.82%, 95% C.I. −1.18; −0.45). Interestingly, a trend for a greater reduction in HbA1c was observed in males compared to females (respectively: −0.86%; 95% C.I. −1.22; −0.51; −0.42%; 95% C.I. −0.72; −0.11; *p* = n.s.). In patients switching from DPP4i to oral semaglutide, a further slight improvement in HbA1c was observed (*n* = 46, −0.46; 95% C.I. −0.6; 0.2, *p* = n.s.).

Conversely, body weight loss did not vary across the different subgroups analyzed.

An improvement in WC, lipid profile and blood pressure was also observed. Notably, WC demonstrated a significant reduction of 2.1 cm (95% C.I. −5.6; 1.1, *p* < 0.05). A substantial decrease was observed in total cholesterol levels (−17.41 mg/dL; 95% C.I. −27.01; −7.80, *p* < 0.0001) and LDL cholesterol (−17.89 mg/dL; 95% C.I. −27.34; −8.44, *p* < 0.0001). Modest and non-significant changes were observed in HDL cholesterol (−0.49 mg/dL; 95% C.I. −4.60; 3.62, *p* = n.s.) and triglyceride levels (−17.20 mg/dL, 95% C.I. −47.09; 12.68, *p* = n.s.). Liver enzymes were in the normal limits and remained unchanged. Furthermore, SBP and DBP demonstrated a non-significant reduction of −3 mmHg (95% C.I. −5.1; 2, *p* = n.s.) and −3 mmHg (95% C.I. −4.8; 1, *p* = n.s.), respectively.

Finally, renal function remained unchanged throughout the observation period [eGFR −0.05 mL/min/m2 (95% C.I. −1.82; 1.99, *p* = n.s.); microalbuminuria −8 mg/L (95% C.I. −27; 49, *p* = n.s.)].

### 3.3. Concomitant Glucose-Lowering Therapy

Baseline glucose-lowering therapy was overall modified as follows: sulfonylureas and DPP4i were uniformly discontinued in all subjects using them. The number of patients treated with pioglitazone, SGLT2i, and insulin remained largely unchanged, as illustrated in Figure 1.

### 3.4. Oral Semaglutide Dosing

The starting dose of 3 mg of oral semaglutide was prescribed to all patients (100%). At the 6-month follow up, 93% (*n* = 130) of patients adhered to the prescribed dosage of 7 mg per day. Only in 2% (*n* = 3) of subjects semaglutide was up-titrated to the maximum dosage of 14 mg per day. In 5% (*n*= 7) of cases the dosage of 3 mg was maintained at 24 weeks follow up. The majority of patients (85%) received a prescription for the pre-breakfast dosing, while the remaining received prescription for pre-dinner dosing. Moreover, patients were provided with specific advice regarding the appropriate drug management.

### 3.5. Safety Endpoints: Adverse Events and Discontinuation Rate

No severe or documented hypoglycemic episodes were reported. During the follow-up period, 40 patients (20.8%) discontinued treatment, with a median time to treatment interruption of 4.04 ± 2.53 months. In 12 out of 40 patients, an early discontinuation (up to 45 days) of oral semaglutide was observed. Overall, the main reasons for discontinuation were: gastrointestinal side effects (26 subjects, 13.5%); limited effectiveness in lowering HbA1c and/or body weight (10 subjects, 5.2%); patient’s decision, pregnancy or no specified reasons accounted for the remaining 2.1% of oral drug discontinuation. No difference in terms of side effects or in percentage of drug discontinuation related to gender were observed.

## 4. Discussion

In a routine clinical setting, patients initiating oral semaglutide for treatment intensification showed suboptimal metabolic control, short diabetes duration and obesity. A clinically significant improvement in HbA1c and body weight was achieved after 6 months of therapy, mainly in patients with a more recent diabetes diagnosis, supporting the use of oral semaglutide in the early phase of the disease.

According to prior observation [15], in this study, a gender-related difference in HbA1c reduction was not observed; however, men experienced a slightly greater HbA1c reduction as compared to women, despite comparable glycemic control at baseline [15].

Noteworthy, improvement of glycemic control was observed across all categories of HbA1c, though a consistent amelioration was evident for subjects with HbA1c higher than 8%. Interestingly, when examining changes in body weight, all patients exhibited comparable weight loss, indicating uniform effectiveness of oral semaglutide irrespective of baseline weight categories. Remarkably, the composite outcome including HbA1c levels ≤ 7% and a weight loss > 5% was achieved by almost 25% of subjects. This achievement underscores the potential synergistic benefits of oral semaglutide targeting both glycemic control and weight in diabetes management. It is crucial to emphasize that the simultaneous reduction in HbA1c and body weight yields a substantial decrease in hard renal and cardiovascular endpoints, supporting a holistic approach to diabetes management [12,16].

Beyond the improvement in glycemic control and body weight, this study highlights a broad spectrum of positive metabolic changes associated with oral semaglutide. In the assessment of cardiovascular risk, it is appropriate to include key parameters, such as lipid profile, waist circumference, eGFR, and blood pressure. These are all integral components in the calculation of the 10-year cardiovascular event risk using the SCORE2-Diabetes algorithm, a widely endorsed tool according to the latest cardiology guidelines of the European Society of Cardiology [17]. The outcomes of this observational study highlight the favorable impact of oral semaglutide on almost all these parameters. Notably, lipid profile and WC showed a significant improvement following 6 months of oral semaglutide therapy; moreover, both blood pressure and microalbuminuria exhibited a trend towards amelioration, while eGFR remained stable. These results emphasize the comprehensive metabolic benefits of oral semaglutide beyond glycemic control and weight loss, which lead to cardiovascular risk modulation and overall metabolic health.

It must be noted that the observed benefits were obtained with a submaximal dosage of oral semaglutide. At the 6-months follow up, nearly all patients were on a 7 mg dosage, and only 2% were on a 14 mg dosage. As per standard clinical practice, no intermediate visits were planned between the initial prescription of the drug and the 24-week therapy period. Thus, the results mirror the effectiveness of oral semaglutide from the first prescription to the follow up at 24 weeks. It should be noted that only those patients (*n* = 3) who increased the dosage to 14 mg had attended an unplanned visit. A non-aggressive titration might be explained by the physician’s perception that a rapid dosage increase was not needed to obtain the HbA1c target or by concerns related to gastrointestinal side effects. Indeed, gastrointestinal side effects were experienced by 13.5% of patients, leading to treatment discontinuation in the 20.8% of cases; thus, gastrointestinal illness represents the primary reason for treatment interruption. The discontinuation rate observed in the present study is slightly higher than that observed in both the PIONEER program and the available observational studies with oral semaglutide [18,19,20,21,22,23,24].

The discontinuation rate observed is also larger than that reported for semaglutide s.c. formulation (9.2%) in our previous observational study [25]; this may be explained by a slightly lower tolerability of the oral formulation. Indeed, the analysis of pharmacokinetics and bioavailability profile following different administration routes suggests a larger interindividual variability with oral as compared to subcutaneous semaglutide administration [26]. This could explain the difference in semaglutide tolerability. Thus, reinforcing dietary advice (e.g., prefer low-fat foods, eat meals slowly and only if actually hungry, consume smaller portions, stop eating in case of feeling of fullness) [27] is extremely necessary in people receiving semaglutide oral formulation. It could be conceived that these recommendations had not been followed by patients included in this cohort, probably because physicians and or dieticians failed to comprehensively highlight their priority. Implementing strategies enabling to keep patients adherent on GLP-1RA therapy is anyhow of crucial importance in order to control cardiovascular risk. As recently reported, discontinuing GLP-1RA treatment is associated with an elevated risk of major cardiovascular events, regardless the history of previous cardiovascular events [28].

The efficacy and safety of oral semaglutide have been assessed in the PIONEER program across the wide spectrum of T2D disease course; oral semaglutide was compared with an active comparator (other GLP-1RAs, SGLT2i, DPP4i) and/or placebo in each of the ten, phase 3, randomized studies [29]. Trying to compare the results of the present work with those of RCT trials, it appears that the relatively small series of data obtained are in line with the RCT observations in terms of both efficacy and safety. Considering other real-world studies on T2D patients, in reference to glycemic control, in the present work, the HbA1c reduction at 6 months (−0.68%) was comparable to that shown in the ENDO2S-RWD [21] (−0.7%HbA1c) and in a recent study by Frazer and colleagues [22] (−0.8%). Limiting the comparison to the Italian evidence available, the present cohort reached the greatest glycemic efficacy (−0.3% and −0.4%) [23,24]; conversely, a more effective glycemic control was reported by the IGNITE study [18] (−0.9%), the PIONEER REAL Switzerland [19] (−0.91%), and in a cohort of Japanese T2D patients [20] (−1.24%). In reference to body weight, we observed a weight loss at 6 months of −2.63 kg. A variability in the extent of weight loss emerged in the literature, with some authors reporting larger reductions (from −3.7 to −4.85 kg) [19,21,24] and others showing a modest efficacy on body weight improvement (from −1.4 4 kg to −2 kg) [20,23]. In some studies, data on weight loss were omitted [18,22]. It is important to clarify that our findings are based on a 24-week follow-up period, during which most patients were taking a 7 mg dose of oral semaglutide. In order to achieve a further improvement in glycemic control and weight loss, it would be advisable to consider increasing the dosage to 14 mg. This adjustment is supported by available evidence, suggesting that a higher dosage may be more effective for these outcomes [2,30].

Several differences emerge comparing the present with other published observational studies. Particularly, as compared to US evidence, a far smaller percentage of patients received treatment with sulfonylureas (5% vs. 30%) [18,22]. In addition, the rate of treatment discontinuation (20.8%) was greater than that of other populations (around 14%) [19,23]. Surprisingly, in two of the published observational research no discontinuations were recorded [20,24]. It should be emphasized that in the two US real-world studies (IGNITE trial and the US administrative database study) no information regarding the discontinuation rates was provided [18,22]. This makes it difficult to discuss the discontinuation rate in different clinical experiences among various geographical areas.

While awaiting the results of the ongoing cardiovascular outcome trial (SOUL trial), and considering the global supply chain challenges for subcutaneous GLP-1RA, the findings discussed in the present study can be valuable in supporting clinical decision-making. It is crucial to consider that achieving a significant weight loss, irrespective of the initial starting body weight [31] is of fundamental importance in reducing cardiovascular risk [32]. In an era where prioritizing cardiorenal risk has become a major priority in prescribing antidiabetes medications, it is important to remember that tight glycemic control obtained in safe, during the early stage of the disease (possibly in the first years after diagnosis), significantly reduces the rate of micro and macrovascular complications as well as cardiovascular death [33]. This is particularly noteworthy since oral semaglutide appears to be an excellent therapeutic option for improving glycemic control, curtailing weight gain, or promoting weight loss and positively modulating almost all modifiable cardiovascular risk factors.

### Strengths and Limitations of This Study

This observational retrospective study based on clinical data sources has strengths and limitations. Among strengths, we include the source of the data, which is not insurance or administrative databases; the presented data derive from patients’ electronic detailed medical records manually revised by authors. The limitations are the relatively short observation period and the marginal number of patients reaching 14 mg of oral semaglutide dose (after 24 weeks). Nevertheless, a significant improvement in both HbA1c and weight loss was obtained despite the suboptimal dose used. Background therapy for glycemic control, which included any antidiabetes agent deemed most appropriate as per routine clinical practice, is another aspect to consider in interpreting the obtained results. Overall, such real-world data can complete and integrate the evidence derived from registrational randomized studies, enabling assessing the transferability of results obtained in a control setting into routine clinical practice.

## 5. Conclusions

In a routine clinical setting, patients initiating oral semaglutide for diabetes treatment intensification showed suboptimal metabolic control, short diabetes duration and obesity. A clinically significant improvement in HbA1c and body weight (even at the 7 mg dose) was achieved mainly in patients with a more recent diabetes diagnosis, supporting the use of oral semaglutide in the early phase of the disease. An effective strategy for achieving glycemic and body weight targets may be titrating the dosage up to 14 mg, provided it is well tolerated. Adequate nutritional counselling and therapy management advice may be able to improve treatment adherence, thereby amplifying the benefits associated with oral semaglutide therapy.

## Figures and Tables

**Table 1:** Characteristics of patients at baseline. Data are shown as the medians (IQR) or mean ± (SD) if variables are normally distributed. Abbreviations: IQR, interquartile range; SD, standard deviation; HbA1c, glycated hemoglobin; FPG, fasting plasma glucose; BMI, body mass index; BP, blood pressure; eGFR, estimated glomerular filtration rate; HDL, high-density lipoprotein; LDL, low-density lipoprotein; AST, aspartate aminotransferase; ALT, alanine aminotransferase; gamma GT, gamma-glutamyl transferase; TIA, transient ischemic attack; PCSK9, proprotein convertase subtilisin/kexin type 9; ACE, angiotensin-converting enzyme; ARBs, angiotensin receptor blockers.

Baseline Characteristics of Subjects with Type 2 Diabetes (*n* = 192)	
Median age (IQR), years	67 (15)
Age < 65, %	42
Age ≥ 65, %	53
Female, %	44
Mean baseline HbA1c (SD), %	7.90 (1.19)
Mean baseline HbA1c (SD), mmol/mol	63 (13)
HbA1c < 7%, %	14
HbA1c < 8%, %	64
HbA1c ≥ 8%, %	36
HbA1c between 7 and 10%, %	81
HbA1c ≥ 10%, %	5
Median FPG (IQR), mg/dL	146 (42.5)
Median FPG (IQR), mmol/L	8.1 (2.4)
Mean body weight (SD), kg	82.47 (17.63)
Mean BMI (SD), kg/m^2^	30.41 (5.86)
BMI < 25 kg/m^2^, %	18
BMI 25–30 kg/m^2^, %	35
BMI ≥ 30 kg/m^2^, %	47
Mean waist circumference (SD), cm	108.9 (13.8)
Mean systolic BP (SD), mmHg	138.3 (18)
Mean diastolic BP (SD), mmHg	76.8 (15)
Median diabetes duration (IQR), years	9 (12)
Diabetes duration < 5 years, %	32
Diabetes duration ≥ 5 years, %	68
Mean creatinine (SD), mg/dL	0.96 (0.29)
Mean microalbuminuria (SD), mg/L	48.85 (103.78)
Mean eGFR (SD), mL/min/1.73m^2^	76.73 (20.55)
eGFR< 60 mL/min/1.73m^2^, %	21
eGFR ≥ 60 mL/min/1.73m^2^, %	79
Mean total cholesterol (SD), mg/dL	180 (43.08)
Mean HDL cholesterol (SD), mg/dL	48.77 (12.97)
Mean tryglicerides (SD), mg/dL	163 (105)
Mean LDL cholesterol (SD), mg/dL	101.73 (38.71)
Mean AST (SD), UI/L	21.9 (11.7)
Mean ALT (SD), UI/L	25.7 (21.4)
Mean gamma-GT, UI/L	28.3 (15.7)
Prescribers: endocrinologists, %	100
**Comorbidities/complications (%)**	
Arterial hypertension	63
Dyslipidemia	65
Microangiopathy	35
Retinopathy	16
Nephropathy	25
Neuropathy	3
Peripheral vasculopathy	6
Macroangiopathy	31
Ischemic cardiopathy	14
Heart failure	4
Stroke TIA	3
**Concomitant Medication (%)**	
Antiplatelet agents	36
Anticoagulants	4
Antihyperuricemic agents	14
Statins	53
Ezetimibe	16
PCSK9 inhibitors	0.52
Omega-3 fatty acids	5
Fibrates	4
ACE inhibitors and ARBs	48
Beta blockers	35
Calcium antagonists	23
Diuretics	23
Alpha blockers	6

**Table 2:** Comparison of differences in HbA1c, body weight and metabolic parameters between baseline and 6 months follow up. Abbreviations: HbA1c, glycated hemoglobin; SD, standard deviation; BP, blood pressure; HDL, high-density lipoprotein; LDL, low-density lipoprotein; eGFR, estimated glomerular filtration rate; AST, aspartate aminotransferase; ALT, alanine aminotransferase; gamma GT, gamma-glutamyl transferase; n.s., not significant.

Parameters	Baseline	Follow Up	Change Mean Difference (95% C.I.)	*p* Value
Mean HbA1c (%) ± SD	7.90 ± 1.19	7.28 ± 0.99	−0.68% (−0.92; −0.43)	<0.0001
Mean Body Weight (kg) ± SD	82.47 ± 17.63	77.90 ± 18.59	−2.63 (−3.37; −1.90)	<0.0001
Mean WC (cm) ± SD	108.9 ± 13.8	106.8 ± 13.8	−2.1 (−5.60; 1.1)	<0.05
Mean Systolic BP (mmHg) ± SD	138.3 ± 18	136 ± 8.9	−3 (−5.10; 2)	n.s.
Mean Diastolic BP (mmHg) ± SD	76.8 ± 15	74 ± 11	−3 (−4.80; 1)	n.s.
Mean Total Cholesterol (mg/dL) ± SD	180 ± 43.08	159.80 ± 36.1	−17.41 (−27.01; −7.80)	<0.0001
Mean HDL Cholesterol (mg/dL) ± SD	48.77 ± 12.97	47.6 ± 14.8	−0.49 (−4.60; 3.62)	n.s
Mean Triglycerides (mg/dL) ± SD	163 ± 105	154 ± 72.8	−17.20 (−47.09; 12.68)	n.s.
Mean LDL Cholesterol (mg/dL) ± SD	101.73 ± 38.71	82.95 ± 32.1	−17.89 (−27.34; −8.44)	<0.0001
Mean eGFR (ml/min/m^2^) ± SD	76.73 ± 20.55	74.5 ± 21.1	−0.05 (−1.82; 1.99)	n.s
Mean Microalbuminuria (mg/L) ± SD	48.85 ± 103.78	42 ± 122	−8 (−27; 49)	n.s.
Mean AST (UI/L) ± SD	21.9 ± 11.7	19.5 ± 7.6	−2.40 (−6.38; 1.50)	n.s.
Mean ALT (UI/L) ± SD	25.7 ± 21.4	22.5 ± 10.4	−3.19 (−9.76; 3.39)	n.s.
Mean Gamma GT (UI/L) ± SD	28.3 ± 15.7	24.3 ± 14	−4 (−1.70; 5.70)	n.s.

## Data Availability

The data presented in this study are available on request from the corresponding author due to privacy reasons.
